# Association between the *TP53* codon 72 polymorphism and risk of oral squamous cell carcinoma in Asians: a meta-analysis

**DOI:** 10.1186/1471-2407-14-469

**Published:** 2014-06-26

**Authors:** Xian-Tao Zeng, Wei Luo, Pei-Liang Geng, Yi Guo, Yu-Ming Niu, Wei-Dong Leng

**Affiliations:** 1 Department of Stomatology and Center for Evidence-Based Medicine and Clinical Research, Taihe Hospital, Hubei University of Medicine, Shiyan 442000, P.R. China; 2 Institute and Department of Stomatology, Chinese PLA General Hospital, Beijing 100853, P.R. China; 3 Department of Oncology, Chinese PLA General Hospital, Beijing 100853, P.R. China; 4 Department of Epidemiology, School of Public Health, Wuhan University, Wuhan 430071, P.R. China

**Keywords:** *TP53* rs1042522, *TP53* codon 72 polymorphism, Oral squamous cell carcinoma, Human papillomavirus, Meta-analysis

## Abstract

**Background:**

Several epidemiological studies have previously investigated the association between the *TP53* codon 72 polymorphism and oral squamous cell carcinoma (OSCC) susceptibility; however, current results are inconsistent. We therefore performed this meta-analysis to thoroughly investigate any association among Asian patients.

**Methods:**

A comprehensive search of PubMed and Embase databases was performed up to December 2013. We only considered studies consisting of patients diagnosed with OSCC by pathological methods. Statistical analyses were performed using Review Manager (RevMan) 5.2 software and odds ratios (ORs) with 95% confidence intervals (CIs) were used to assess the association.

**Results:**

A total of 11 case–control studies involving 2,298 OSCC patients and 2,111 controls were included. We found no association between the *TP53* codon 72 polymorphism and OSCC susceptibility [(OR = 0.77, 95% CI = 0.48–1.22) for Arg vs. Pro; (OR = 0.67, 95% CI = 0.31–1.43) ArgArg vs. ProPro; (OR = 1.14, 95% CI = 0.97–1.35) ArgPro vs. ProPro; (OR = 0.85, 95% CI = 0.53–1.34) (ArgPro + ArgArg) vs. ProPro; or (OR = 0.34, 95% CI = 0.34–1.23) for ArgArg vs. (ProPro + ArgPro)]. However, subgroup analysis demonstrated an association between the *TP53* codon 72 polymorphism and human papillomavirus (HPV)-related OSCC patients. Although statistical heterogeneity was detected, there was no evidence of publication bias.

**Conclusions:**

Current results suggest that the *TP53* codon 72 polymorphism is not associated with OSCC in Asians without the presence of HPV infection. Further research is necessary to determine if such a relationship exists in HPV-related OSCC patients.

## Background

Oral cancer is ranked as the 11th most common type of cancer worldwide [1], with a higher prevalence in South and Southeast Asian countries such as India, Bangladesh, China, and Sri Lanka [2]. Oral squamous cell carcinoma (OSCC) originates from the squamous cells that cover the surface of the mouth and is a major type of oral cancer, accounting for more than 90% of cases [3]. Tobacco use (chewing with or without smoking), alcohol consumption, and human papillomavirus (HPV) infection are important risk factors for development of OSCC [4,5]; however, molecular mechanisms relating to OSCC are still being investigated, while genetic predisposition is gaining increasing attention [6-8].

The tumor protein p53 (*TP53*) gene, located on chromosome 17p13, is one of the most frequently mutated genes in human cancers and has been reported to be a significant determining factor in carcinogenesis [9]. The codon 72 polymorphism (rs1042522) is located in exon 4 of TP53 gene, and involves a CCC → CGC transition leading to a proline (Pro) → arginine (Arg) amino acid substitution at position 72 (Pro72Arg) (http://www.ncbi.nlm.nih.gov/snp/?term=rs1042522) [10]. Many published meta-analyses have indicated that the *TP53* codon 72 polymorphism might be associated with increased susceptibility to cervical cancer [11], bladder cancer [12], and nasopharyngeal carcinoma [13].

Several previous studies have explored the association between the *TP53* codon 72 polymorphism and OSCC susceptibility; however, existing results are inconsistent. In 2009, Zhuo et al. performed a meta-analysis of nine case–control studies and found that the *TP53* codon 72 polymorphism might be a risk factor for oral carcinoma [14]. This is in agreement with another meta-analysis of 17 case–control studies by Jiang et al. published in 2013 [15]. Both meta-analyses included patients with OSCC but did not stratify the condition as a separate subgroup [14,15]. Additionally, several more recent studies have since been published. Therefore, we conducted this meta-analysis to obtain accurate and up-to-date estimates of the association between the *TP53* codon 72 polymorphism and OSCC susceptibility in Asians. Subgroup analysis was also performed to investigate any potential HPV-specific effects.

## Methods

This meta-analysis adheres to the recommended Preferred Reporting Items for Systematic Reviews and Meta-Analyses (PRISMA) guidelines [16].

### Inclusion criteria

We included case–control studies that met the following eligibility criteria: (1) evaluated the association between the *TP53* codon 72 polymorphism and OSCC susceptibility in Asians; (2) included OSCC cases diagnosed by histologic methods or clearly reported the type, and contained healthy or cancer-free controls; (3) provided the number of individual genotypes in both the case and control groups, or enabled the genotypes to be calculated from available published data; (4) published in English or Chinese; and (5) used genotyping was polymerase chain reaction (PCR) including PCR- polymerase chain reaction-restriction fragment length polymorphism (RFLP) and PCR- polymerase chain reaction-single strand conformation polymorphism (SSCP) for genotyping.

### Search strategy

We searched PubMed and Embase databases up to December 10, 2013 with the following search items: [(oral OR tongue OR mouth) AND (cancer OR carcinoma) AND (p53 OR TP53) AND polymorphism]. Reference lists of the included studies and published meta-analyses on related topics were also screened for additional studies.

### Data extraction

Two authors independently extracted the following trial data from included studies: last name of the first author, publication year, countries of origin, HPV status of cases, source of control, number and genotyping distribution of cases and controls, diagnostic method for OSCC, genotyping method, and Hardy-Weinberg Equilibrium (HWE) for controls [17]. Disagreements were resolved by discussion.

### Statistical analysis

We employed the fixed-effect analytical model first to pool results of the included studies, and the *I*^2^ statistic [18] was used to test for statistical heterogeneity. If *I*^2^ was more than 40%, we switched to a random-effects model. The odds ratios (ORs) and relevant 95% confidence intervals (CIs) were used to quantify the strength of association between the *TP53* codon 72 polymorphism and OSCC susceptibility using five genetic models: Arg vs. Pro, ArgArg vs. ProPro, ArgPro vs. ProPro, (ArgPro + ArgArg) vs. ProPro, and ArgArg vs. (ProPro + ArgPro). Additionally, subgroups analyses based on HPV status, source of controls, and HWE status for controls were performed. Publication bias was detected by examination of funnel plots. All statistical analyses were conducted using Review Manager (RevMan) software (version 5.2 for Windows).

## Results

### Study characteristics

**Figure 1:**
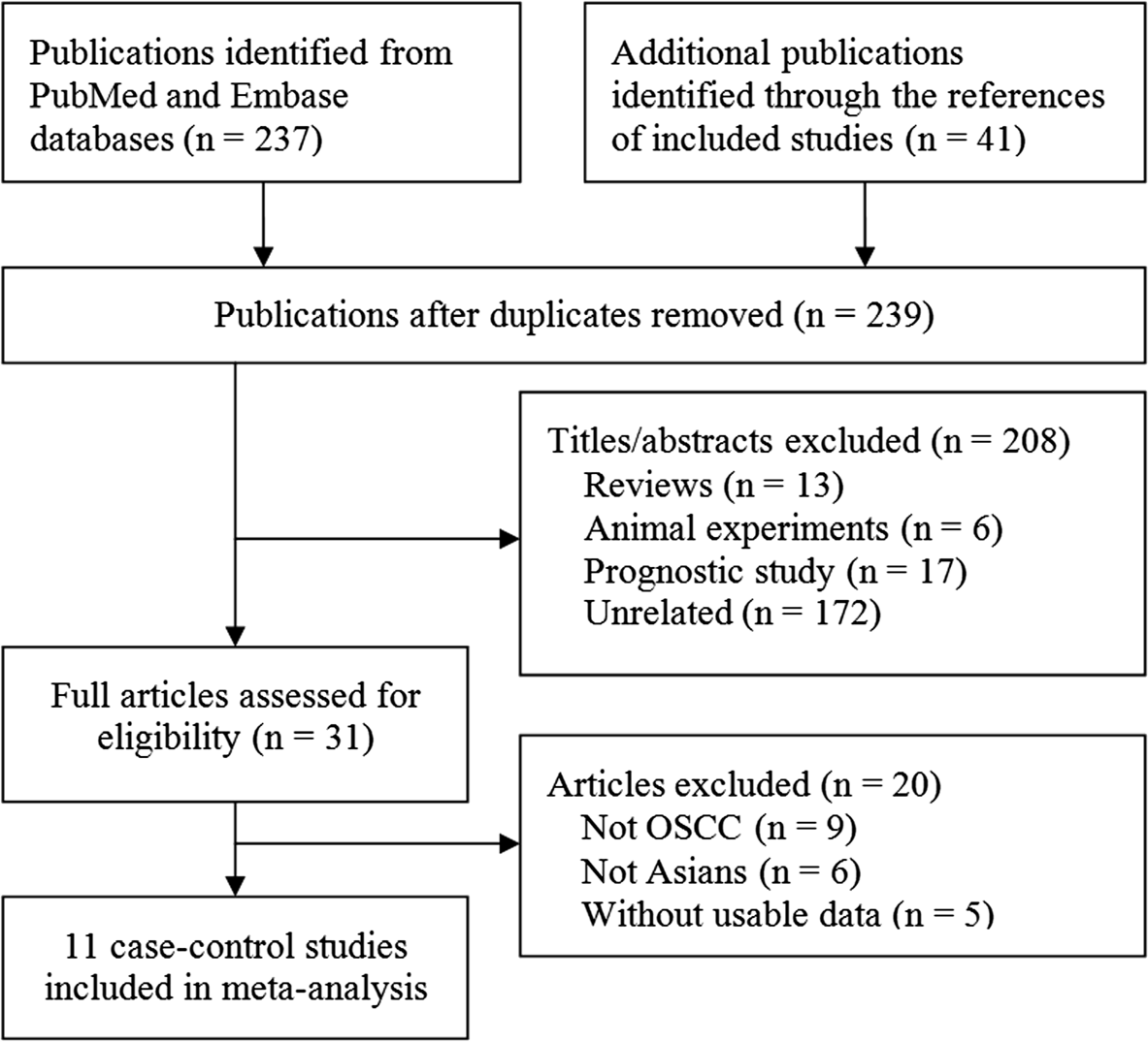
Study selection flow chart.

Our systematic literature search identified 278 studies that met the inclusion criteria. After deduplication and exclusion of the clearly irrelevant studies, we eventually included 11 case–control studies [19-29] involving 2,298 OSCC patients and 2,111 controls. Figure 1 shows the study selection process. Of the 11 included studies, two recruited OSCC patients with HPV [20,28], and three enrolled patients with disrupted HWE [19,21,29]. Baseline characteristics of the 11 studies are summarized in Table 1.

**Table 1:** Characteristics of included studies

**Reference**	**Country**	**OSCC**					**Diagnostic method**	**Source of control**	**Control**				**Genotype method**	**HWE**
		**HPV**	**Total**	**ProPro**	**ArgPro**	**ArgArg**			**Total**	**ProPro**	**ArgPro**	**ArgArg**		
Tandle 2001 [19]	India	No	72	14	52	6	Histopathological	PB	153	31	100	22	PCR	<0.001
Nagpal 2002 [20]	India	Yes	110	21	58	31	Histological	PB	26	2	11	13	PCR	0.876
Kietthubthew 2003 [21]	Thailand	No	97	21	44	32	Histological	PB	97	28	34	35	PCR	0.004
Hsieh 2005 [22]	China	No	629	114	328	187	Histological	PB	371	66	177	128	PCR-RFLP	0.723
Kuroda 2007 [24]	Japan	No	100	15	44	41	Histological	HB	271	45	117	109	PCR-RFLP	0.159
Bau 2007 [23]	China	No	137	21	70	46	NA	HB	105	22	65	18	PCR	0.139
Lin 2008 [25]	China	No	297	46	155	96	Histological	PB	280	52	156	72	PCR-RFLP	0.085
Tu 2008 [26]	China	No	189	30	106	53	NA	HB	116	15	60	41	PCR	0.337
Misra 2009 [27]	India	No	308	66	155	87	Histopathological	HB	342	98	159	85	PCR	0.203
Saini 2011 [28]	Malaysia	Yes	99	37	40	22	NA	HB	90	23	39	28	PCR	0.215
Saleem 2013 [29]	Pakistan	No	260	125	113	22	NA	PB	260	33	23	204	PCR-SSCP	<0.001

OSCC, oral squamonus cell carcinoma; HPV, human papillomavirus; NA, not available; HB, hospital-based; PB, population-based; HWE, Hardy Weinberg Equilibrium.

### Meta-analysis

**Figure 2:**
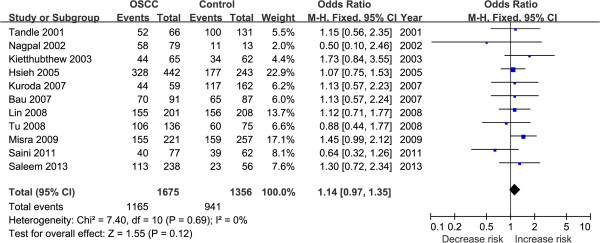
**Forest plot.** This represents the OSCC risk associated with the *TP53* codon 72 polymorphism in Asians for the ArgPro vs. ProPro genetic model.

Table 2 illustrates results of the overall and subgroup analyses. Overall, there was no association between the *TP53* codon 72 polymorphism and OSCC susceptibility in Asians [(OR = 0.77, 95% CI = 0.48–1.22) for Arg vs. Pro; (OR = 0.67, 95% CI = 0.31–1.43) for ArgArg vs. ProPro; (OR = 1.14, 95% CI = 0.97–1.35) for ArgPro vs. ProPro, Figure 2; (OR = 0.85, 95% CI = 0.53–1.34) for (ArgPro + ArgArg) vs. ProPro; and (OR = 0.34, 95% CI = 0.34–1.23) for ArgArg vs. (ProPro + ArgPro)].

**Table 2:** Overall and subgroups meta-analysis of TP53 codon 72 polymorphism and OSCC risk in Asians

	**N**	**Arg vs. Pro**		**ArgArg vs. ProPro**		**ArgPro vs. ProPro**		**(ArgPro + ArgArg) vs. ProPro**		**ArgArg vs. (ProPro + ArgPro)**	
		**OR (95% CI)**	**I**^**2**^**(%)**	**OR (95% CI)**	**I**^**2 **^**(%)**	**OR (95% CI)**	**I**^**2 **^**(%)**	**OR (95% CI)**	**I**^**2 **^**(%)**	**OR (95% CI)**	**I**^**2 **^**(%)**
Overall	11	0.77(0.48-1.22)	96	0.67 (0.31-1.43)	94	1.14 (0.97-1.35)	0	0.85 (0.53-1.34)	87	0.64 (0.34-1.23)	95
HPV status											
Without	9	0.81 (0.48-1.39)	97	0.75 (0.32-1.79)	95	1.20 (1.01-1.43)	0	0.93 (0.56-1.55)	89	0.68 (0.32-1.42)	96
With	2	0.60 (0.43-0.85)	0	0.41 (0.21-0.81)	0	0.61 (0.33-1.14)	0	0.54 (0.30-0.96)	0	0.54 (0.32-0.91)	0
Source of controls											
PB	7	0.60 (0.26-1.38)	98	0.43 (0.11-1.64)	96	1.15 (0.92-1.44)	0	0.71 (0.33-1.55)	92	0.37 (0.12-1.19)	96
HB	5	1.03 (0.79-1.35)	71	1.09 (0.64-1.87)	69	1.13 (0.87-1.45)	17	1.06 (0.73-1.53)	52	1.22 (0.88-1.69)	59
HWE											
>0.05	8	0.99 (0.82-1.20)	70	1.03 (0.70-1.50)	65	1.10 (0.91-1.32)	0	1.08 (0.91-1.29)	38	0.98 (0.73-1.31)	69
<0.05	3	0.44 (0.08-2.44)	99	0.27 (0.02-3.72)	97	1.36 (0.92-2.00)	0	0.61 (0.13-2.82)	95	0.23 (0.02-2.70)	98

OSCC, oral squamonus cell carcinoma; HPV, human papillomavirus; HB, hospital-based; PB, population-based; HWE, Hardy Weinberg Equilibrium.

Results of the subgroup analyses stratified by source of controls and HWE status for controls were similar to those of the overall analyses. However, when stratified by HPV status, a correlation between the *TP53* codon 72 polymorphism and HPV infection was observed (Table 2).

### Publication bias

**Figure 3:**
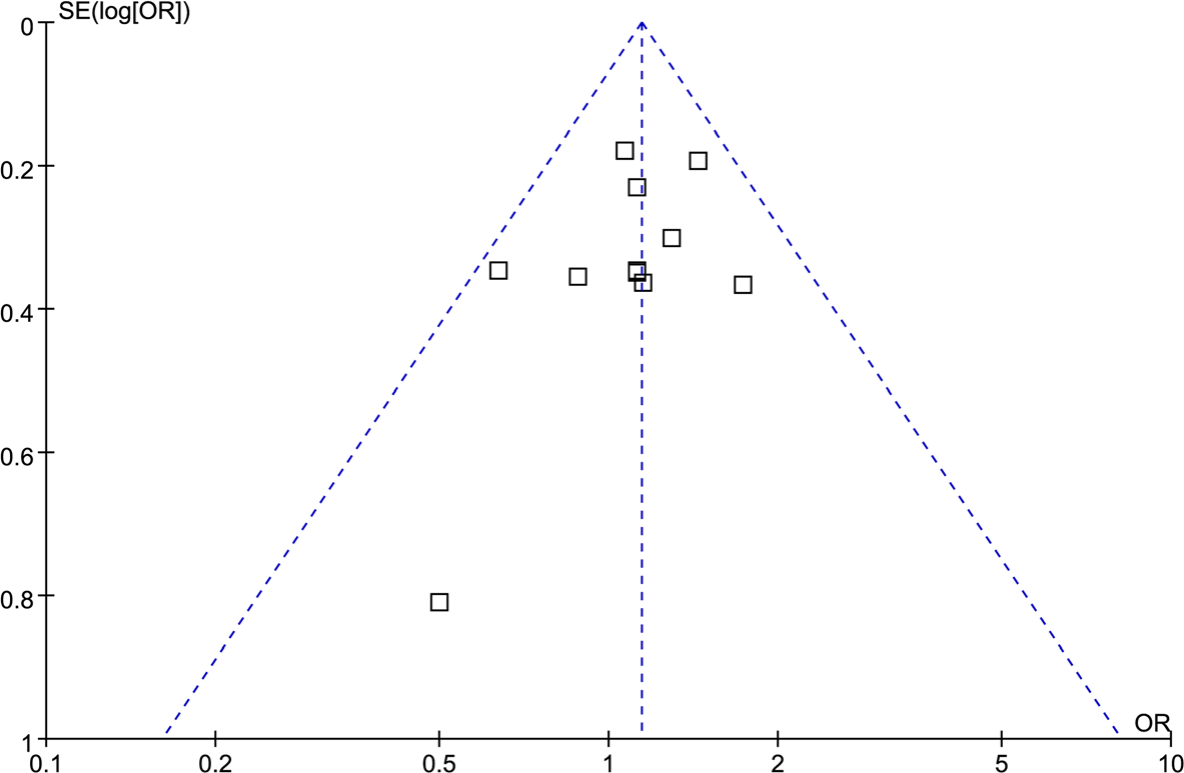
**Funnel plot.** This represents the publication bias test based on the ArgPro vs. ProPro genetic model.

A funnel plot based on the ArgPro vs. ProPro genetic model showed a relatively symmetrical distribution, enabling us to conclude that there was no publication bias (Figure 3).

## Discussion

Arg and Pro are two distinct functional alleles that are encoded by the *TP53* codon 72, and Pro to Arg is the most informative polymorphism in the *TP53* gene and have been found to be associated with human cancers [10,30]. Among the published meta-analyses exploring the association between this polymorphism and cancers, some revealed an increased susceptibility of disease [11-13], while others failed to find any association [31-33]. Results from these meta-analyses indicate an interesting phenomenon, which is that different meta-analyses of the same cancer type could yield opposite results. Although two meta-analyses investigating the relationship of the *TP53* codon 72 polymorphism and oral cancer susceptibility both reached the same conclusions [14,15], such association among the Asian population is unclear. Given that OSCC has a high incidence in this population, we conducted the current meta-analysis to further investigate if the *TP53* codon 72 polymorphism plays a role in the development of OSCC.

A total of 2,298 OSCC patients and 2,111 controls were included in our meta-analysis. Results of the overall population demonstrated a negative association of the *TP53* codon 72 polymorphism and OSCC, although subgroup analysis revealed a positive correlation between the polymorphism and HPV status in OSCC patients. Our results are in contrast with those reported by Zhou et al. [14], which was based on three studies reporting HPV infection status; however, only one of these focused on an Asian population [20]. Moreover, this earlier meta-analysis is limited by its small sample size and mixed ethnicity. In contrast to the two previous meta-analyses [14,15], our meta-analysis only focused on OSCC in Asians.

The relationship between HPV and OSCC has been previously established [34]. Our meta-analysis also found that the *TP53* codon 72 polymorphism was associated with HPV-related OSCC susceptibility cases. However, because there is no association between this polymorphism and non-HPV OSCC cases, it is currently unclear whether the polymorphism is merely a marker of HPV-related OSCC. Further research is warranted to investigate this relationship.

In 2011, Heah et al. found a significant correlation between p53 expression and *TP53* aberration in 26 OSCC cases [35]. This finding is in contrast to the results of our present meta-analysis, although it should be noted that *TP53* contains multiple polymorphisms in addition to the one in codon 72.

Our meta-analysis has a number of limitations. First, like all meta-analyses, it is a secondary retrospective study that is limited by various factors including quality of the original studies, study population differences, and the measurement tools used. Second, statistical heterogeneity is substantial, although this is extremely common in meta-analyses of genetic association studies. We therefore performed subgroup analyses to consider the factors that may have contributed to the high degree of heterogeneity. Third, our included studies lacked comprehensive genotype information so the results of our meta-analysis were analyzed using unadjusted data; hence, we could not generate a more accurate analysis based on other adjusted factors. Finally, the sample size of our meta-analysis is relatively small and studies published in languages other than Chinese and English were not considered for inclusion.

## Conclusions

Our meta-analysis showed a lack of association between the *TP53* codon 72 polymorphism and OSCC susceptibility in Asians, although subgroup analysis demonstrated an association between the polymorphism and HPV-related OSCC patients. Because of the numerous limitations of this meta-analysis including small sample size and substantial statistical heterogeneity, our results should be interpreted with caution and further data from high-quality, well-conducted clinical studies of adequate statistical power are needed.

## Abbreviations

Arg: Arginine; CI: Confidence interval; HPV: Human papillomavirus; HWE: Hardy-Weinberg Equilibrium; OR: Odds ratio; OSCC: Oral squamous cell carcinoma; PCR: Polymerase chain reaction; PRISMA: Preferred Reporting Items for Systematic Reviews and Meta-Analyses; Pro: Proline; RFLP: Restriction fragment length polymorphism; SSCP: Single strand conformation polymorphism; TP53: Tumor protein p53.

## Competing interests

The authors declare that they have no competing interests.

## Authors’ contributions

XTZ and YMN extracted the data and wrote the manuscript. WL and PLG performed statistical analysis. XTZ and WL carried out the systematic literature search and data collection. YG and WDL reviewed the manuscript. All authors approved the final manuscript.

## Pre-publication history

The pre-publication history for this paper can be accessed here:

http://www.biomedcentral.com/1471-2407/14/469/prepub

## References

[B1] Warnakulasuriya S Global epidemiology of oral and oropharyngeal cancer Oral Oncol 2009 45 4–5 309 316 18804401 10.1016/j.oraloncology.2008.06.002

[B2] Ghani WM Doss JG Jamaluddin M Kamaruzaman D Zain RB Oral Cancer Awareness and its Determinants among a Selected Malaysian Population Asian Pac J Cancer Prev 2013 14 3 1957 1963 23679299 10.7314/apjcp.2013.14.3.1957

[B3] Brinkman BM Wong DT Disease mechanism and biomarkers of oral squamous cell carcinoma Curr Opin Oncol 2006 18 3 228 233 16552233 10.1097/01.cco.0000219250.15041.f8

[B4] Goot-Heah K Kwai-Lin T Froemming GR Abraham MT Nik Mohd Rosdy NM Zain RB Human papilloma virus 18 detection in oral squamous cell carcinoma and potentially malignant lesions using saliva samples Asian Pac J Cancer Prev 2012 13 12 6109 6113 23464414 10.7314/apjcp.2012.13.12.6109

[B5] Xia LY Zeng XT Li C Leng WD Fan MW Association between p53 Arg72Pro polymorphism and the risk of human papillomavirus-related head and neck squamous cell carcinoma: a meta-analysis Asian Pac J Cancer Prev 2013 14 10 6127 6130 24289637 10.7314/apjcp.2013.14.10.6127

[B6] Niu YM Shen M Li H Ni XB Zhou J Zeng XT Leng WD Wu MY No association between MTHFR A1298C gene polymorphism and head and neck cancer risk: a meta-analysis based on 9,952 subjects Asian Pac J Cancer Prev 2012 13 8 3943 3947 23098497 10.7314/apjcp.2012.13.8.3943

[B7] Niu Y Hu Y Wu M Jiang F Shen M Tang C Chen N CYP2E1 Rsa I/Pst I polymorphism contributes to oral cancer susceptibility: a meta-analysis Mol Biol Rep 2012 39 1 607 612 21553050 10.1007/s11033-011-0777-3

[B8] Taniyama Y Takeuchi S Kuroda Y Genetic polymorphisms and oral cancer J UOEH 2010 32 3 221 236 20857816 10.7888/juoeh.32.221

[B9] Tsui IF Poh CF Garnis C Rosin MP Zhang L Lam WL Multiple pathways in the FGF signaling network are frequently deregulated by gene amplification in oral dysplasias Int J Cancer 2009 125 9 2219 2228 19623652 10.1002/ijc.24611 PMC2761835

[B10] Ara S Lee PS Hansen MF Saya H Codon 72 polymorphism of the TP53 gene Nucleic Acids Res 1990 18 16 4961 1975675 10.1093/nar/18.16.4961 PMC332028

[B11] Zhou X Gu Y Zhang SL Association between p53 codon 72 polymorphism and cervical cancer risk among Asians: a HuGE review and meta-analysis Asian Pac J Cancer Prev 2012 13 10 4909 4914 23244080 10.7314/apjcp.2012.13.10.4909

[B12] Xu T Xu ZC Zou Q Yu B Huang XE P53 Arg72Pro polymorphism and bladder cancer risk–meta-analysis evidence for a link in Asians but not Caucasians Asian Pac J Cancer Prev 2012 13 5 2349 2354 22901221 10.7314/apjcp.2012.13.5.2349

[B13] Zhuo XL Cai L Xiang ZL Zhuo WL Wang Y Zhang XY TP53 codon 72 polymorphism contributes to nasopharyngeal cancer susceptibility: a meta-analysis Arch Med Res 2009 40 4 299 305 19608020 10.1016/j.arcmed.2009.03.006

[B14] Zhuo XL Li Q Zhou Y Cai L Xiang ZL Yuan W Zhang XY Study on TP53 codon 72 polymorphisms with oral carcinoma susceptibility Arch Med Res 2009 40 7 625 634 20082880 10.1016/j.arcmed.2009.09.004

[B15] Jiang N Pan J Wang L Duan YZ No significant association between p53 codon 72 Arg/Pro polymorphism and risk of oral cancer Tumour Biol 2013 34 1 587 596 23192640 10.1007/s13277-012-0587-9

[B16] Moher D Liberati A Tetzlaff J Altman DG Group P Preferred reporting items for systematic reviews and meta-analyses: the PRISMA statement BMJ 2009 339 b2535 19622551 10.1136/bmj.b2535 PMC2714657

[B17] Salanti G Amountza G Ntzani EE Ioannidis JP Hardy-Weinberg equilibrium in genetic association studies: an empirical evaluation of reporting, deviations, and power Eur J Hum Genet 2005 13 7 840 848 15827565 10.1038/sj.ejhg.5201410

[B18] Huedo-Medina TB Sanchez-Meca J Marin-Martinez F Botella J Assessing heterogeneity in meta-analysis: Q statistic or I2 index? Psychol Methods 2006 11 2 193 206 16784338 10.1037/1082-989X.11.2.193

[B19] Tandle AT Sanghvi V Saranath D Determination of p53 genotypes in oral cancer patients from India Br J Cancer 2001 84 6 739 742 11259085 10.1054/bjoc.2000.1674 PMC2363816

[B20] Nagpal JK Patnaik S Das BR Prevalence of high-risk human papilloma virus types and its association with P53 codon 72 polymorphism in tobacco addicted oral squamous cell carcinoma (OSCC) patients of Eastern India Int J Cancer 2002 97 5 649 653 11807792 10.1002/ijc.10112

[B21] Kietthubthew S Sriplung H Au WW Ishida T The p53 codon 72 polymorphism and risk of oral cancer in Southern Thailand Asian Pac J Cancer Prev 2003 4 3 209 214 14507241

[B22] Hsieh LL Huang TH Chen IH Liao CT Wang HM Lai CH Liou SH Chang JT Cheng AJ p53 polymorphisms associated with mutations in and loss of heterozygosity of the p53 gene in male oral squamous cell carcinomas in Taiwan Br J Cancer 2005 92 1 30 35 15583690 10.1038/sj.bjc.6602271 PMC2361746

[B23] Bau DT Tsai MH Lo YL Hsu CM Tsai Y Lee CC Tsai FJ Association of p53 and p21(CDKN1A/WAF1/CIP1) polymorphisms with oral cancer in Taiwan patients Anticancer Res 2007 27 3B 1559 1564 17595776

[B24] Kuroda Y Nakao H Ikemura K Katoh T Association between the TP53 codon72 polymorphism and oral cancer risk and prognosis Oral Oncol 2007 43 10 1043 1048 17306604 10.1016/j.oraloncology.2006.12.001

[B25] Lin YC Huang HI Wang LH Tsai CC Lung O Dai CY Yu ML Ho CK Chen CH Polymorphisms of COX-2–765G > C and p53 codon 72 and risks of oral squamous cell carcinoma in a Taiwan population Oral Oncol 2008 44 8 798 804 18234542 10.1016/j.oraloncology.2007.10.006

[B26] Tu HF Chen HW Kao SY Lin SC Liu CJ Chang KW MDM2 SNP 309 and p53 codon 72 polymorphisms are associated with the outcome of oral carcinoma patients receiving postoperative irradiation Radiother Oncol 2008 87 2 243 252 18423915 10.1016/j.radonc.2008.03.018

[B27] Misra C Majumder M Bajaj S Ghosh S Roy B Roychoudhury S Polymorphisms at p53, p73, and MDM2 loci modulate the risk of tobacco associated leukoplakia and oral cancer Mol Carcinog 2009 48 9 790 800 19204927 10.1002/mc.20523

[B28] Saini R Tang TH Zain RB Cheong SC Musa KI Saini D Ismail AR Abraham MT Mustafa WM Santhanam J Significant association of high-risk human papillomavirus (HPV) but not of p53 polymorphisms with oral squamous cell carcinomas in Malaysia J Cancer Res Clin Oncol 2011 137 2 311 320 20419384 10.1007/s00432-010-0886-8 PMC11828103

[B29] Saleem S Azhar A Hameed A Khan MA Abbasi ZA Qureshi NR Ajmal M P53 (Pro72Arg) polymorphism associated with the risk of oral squamous cell carcinoma in gutka, niswar and manpuri addicted patients of Pakistan Oral Oncol 2013 49 8 818 823 23683469 10.1016/j.oraloncology.2013.04.004

[B30] Hollstein M Sidransky D Vogelstein B Harris CC p53 mutations in human cancers Science 1991 253 5015 49 53 1905840 10.1126/science.1905840

[B31] Sousa H Santos AM Pinto D Medeiros R Is the p53 codon 72 polymorphism a key biomarker for cervical cancer development? A meta-analysis review within European populations Int J Mol Med 2007 20 5 731 741 17912468

[B32] Matakidou A Eisen T Houlston RS TP53 polymorphisms and lung cancer risk: a systematic review and meta-analysis Mutagenesis 2003 18 4 377 385 12840112 10.1093/mutage/geg008

[B33] Zhou Y Li N Zhuang W Liu GJ Wu TX Yao X Du L Wei ML Wu XT P53 codon 72 polymorphism and gastric cancer: a meta-analysis of the literature Int J Cancer 2007 121 7 1481 1486 17546594 10.1002/ijc.22833

[B34] Kreimer AR Clifford GM Boyle P Franceschi S Human papillomavirus types in head and neck squamous cell carcinomas worldwide: a systematic review Cancer Epidemiol Biomarkers Prev 2005 14 2 467 475 15734974 10.1158/1055-9965.EPI-04-0551

[B35] Heah KG Hassan MI Huat SC p53 Expression as a marker of microinvasion in oral squamous cell carcinoma Asian Pac J Cancer Prev 2011 12 4 1017 1022 21790244

